# Developing machine learning models to improve cardiovascular risk prediction for people living with HIV

**DOI:** 10.1016/j.ijcrp.2026.200683

**Published:** 2026-07-18

**Authors:** Hari Dandapani, Yi-Yun Chen, Michael Kwok, Vrishali Lopes, Christopher Halladay, Gerald S. Bloomfield, Christoper T. Longenecker, Jennifer L. Sullivan, Gaurav Choudhary, James L. Rudolph, Wen-Chih Wu, Sebhat Erqou

**Affiliations:** a Department of Medicine, Alpert Medical School of Brown University, Providence, RI, USA; b Lifespan Cardiovascular Institute, Rhode Island Hospital, Providence, RI, USA; c Department of Medicine, Washington University School of Medicine, St. Louis, MI, USA; d Research Services, VA Providence Healthcare System, Providence, RI, USA; e Department of Medicine, Duke Global Health Institute and Duke Clinical Research Institute, Duke University, Durham, NC, USA; f Department of Medicine, University of Washington, Seattle, WA, USA; g Department of Health Services, Policy and Practice, Brown University School of Public Health, Providence, RI, USA; h Department of Medicine, VA Providence Healthcare System, Providence, RI, USA; i Division of Cardiology, Mary Washington Hospital, Fredericksburg, VA, USA

**Keywords:** Atherosclerotic cardiovascular disease, Risk prediction, Machine learning, Artificial intelligence, HIV

## Abstract

**Background:**

As life expectancy rises for people with HIV, atherosclerotic cardiovascular disease (ASCVD) has become a major contributor to morbidity. Extant risk models understate this risk, stressing the need for better models for HIV patients.

**Methods:**

We studied new ASCVD events using Veterans Health Administration data (baseline 2010-15 and follow-up through 2020). We built four machine learning (ML) models to predict CVD: K-nearest neighbors, Random Forest, Logistic Regression and Neural Network, which were compared to two general risk models: Framingham Risk Score (FRS) and Pooled Cohort Equations (PCE). ML models were trained on all Veterans and only on HIV-positive Veterans and assessed with 5-fold validation. We measured discrimination via area under the receiver operating characteristic curve (AUC) and calibration via Hosmer-Lemeshow.

**Results:**

20,650 Veterans with HIV and 102,654 without HIV were included. HIV patients were 97% male and 51% Black, with a mean age of 52 years. Models trained on all data had better discrimination than models trained only on HIV data. Neural Network and Logistic Regression models trained on all data, and both Random Forest models, had moderately improved discrimination compared to FRS and PCE (AUC ∼0.70 for ML models vs. ∼0.65). FRS and PCE underpredicted CVD risk with observed-to-expected ratios of 2.1 and 1.7, while ML models had ratios closer to 1.

**Conclusions:**

ML models for CVD risk can enhance predictive performance in HIV, with a notable impact on underprediction. Models developed in HIV and non-HIV mixed populations have the best performance.

## Highlights

• Machine learning models improve ASCVD risk prediction in people with HIV.
• Machine learning models demonstrate better calibration than standard risk scores.
• Neural Network and Random Forest models showed the highest performance.
• Models developed using mixed HIV/non-HIV training data showed highest performance.
• Traditional cardiac risk factors maintain the highest value in risk prediction in HIV.

## Graphical abstract

**Figure:**
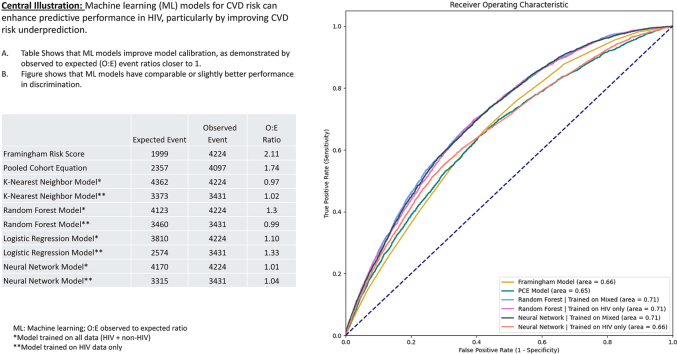

## 1 Background

Cardiovascular disease (CVD) has become a leading cause of morbidity and mortality among people living with HIV (PLHIV), surpassing AIDS-related illnesses in many high-resource settings [1]. This elevated risk is believed to be due to a combination of traditional risk factors, psychosocial risk factors, chronic immune activation, systemic inflammation, and the metabolic consequences of long-term antiretroviral therapy (ART) exposure [2,3].

Therefore, CVD prevention strategies, including statin therapy and risk factor modification, are important for PLHIV. Accurate identification of individuals at elevated CVD risk is essential to ensure timely and targeted preventive interventions. However, accumulating evidence suggests that existing risk prediction tools, including the Framingham Risk Score (FRS) and the Pooled Cohort Equations (PCE), underestimate ASCVD risk in PLHIV [4]. Even HIV-adapted tools such as the Data Collection on Adverse Events of Anti-HIV Drugs Cohort (D:A:D), which includes CD4 count and HIV viral load, offer only marginal improvements over the traditional models and still underestimate risk [4]. While the American College of Cardiology/American Heart Association (ACC/AHA) guidelines acknowledge HIV as a “risk-enhancing factor,” they stop short of offering quantitative guidance, leaving clinicians without a reliable stratification strategy tailored to this population [5].

These limitations underscore the need for novel approaches that can accommodate the complex and nonlinear interactions between traditional and HIV-specific risk factors. Machine learning (ML) models have emerged as promising alternatives due to their ability to learn from high-dimensional data and detect subtle, synergistic patterns that may be overlooked by conventional algorithms. In this project, we used electronic health record (EHR) data from the Veterans Health Administration (VHA) system to develop and compare a range of ML-based risk prediction models for CVD in PLHIV. We evaluated their performance relative to established models, focusing on their ability to improve discrimination, mitigate underprediction, and more accurately reflect cardiovascular risk in this high-risk population.

## 2 Methods

### 2.1 Overview

This is a retrospective cohort study of Veterans with HIV and no prior history of atherosclerotic cardiovascular disease (ASCVD) who received HIV care or primary care within the VHA system from January 2010 to December 2015*,* and a matching set of HIV-negative Veteran controls who received primary care within the same VA facility the same year as the HIV cases. Participants were followed until the first ASCVD diagnosis, death, or study censor date on December 31, 2020. Data were obtained from the VA Corporate Data Warehouse (CDW) using the VA Informatics and Computing Infrastructure (VINCI). The study was approved by the VA Providence Healthcare System Institutional Review Board.

### 2.2 Study population

Veterans with HIV diagnoses were identified using relevant International Classification of Diseases, Ninth Revision (ICD-9), and International Classification of Diseases, Tenth Revision (ICD-10), codes for asymptomatic or symptomatic HIV infection or AIDS recorded during or any time prior to the study period, using a previously validated approach [6,7]. Veterans with HIV must have received care in at least one outpatient primary care clinic or infectious disease/HIV clinic visit during the study baseline period from January 2010 to December 2015 to be included. Matching controls were selected among Veterans without HIV who received care in the same VA facility during the same year as the HIV cases, matched in a 1:6 ratio by age (within 2-year strata), sex, medical center of VA service, and year of service.

### 2.3 Cohort eligibility

Study participants were restricted to ages of 20 and 79 years, regardless of HIV status. Patients who had previous diagnosis of ASCVD were excluded. Patients who were missing any input or outcome variables were also excluded from use in model training and testing. Participants were followed from the date of first primary care or infectious disease clinic visit between January 1, 2010, and December 31, 2015, to incident event date or censor date on December 31, 2020.

### 2.4 Predictor variables

Variables were derived from de-identified EHR data. The dataset comprised 70 input variables for all patients, including demographics, lipid panel results, vital signs, other comorbid medical conditions, and medication use. Laboratory data was obtained from tests performed within 6 months of the baseline date. Adult height was obtained from measurement recorded at any time point within the medical record. For CD4 count and Viral load available values closest to the baseline date were obtained. For patients with HIV, additional HIV-specific variables included CD4 count, viral load, antiretroviral therapy (ART) treatment status, and whether the patient was taking a protease inhibitor, which are known to increase risk of dyslipidemia. Medication use was evaluated as a binary exposure defined as any recorded use during the study observation window.

### 2.5 Outcome variables

The primary ASCVD outcome was total cardiovascular disease incidence (TCVD). For our study, this included incident coronary artery disease (CAD), ischemic cerebrovascular disease (ICVD), or peripheral artery disease (PAD) during follow-up. This was defined based on ICD-10 or Current Procedure Terminology (CPT) codes related to CAD, ICVD, or PAD. The major CAD, ICVD, and PAD procedures considered were coronary artery bypass grafting (CABG), percutaneous coronary interventions, carotid endarterectomy, carotid artery stenting, and peripheral artery angioplasties and stents. The secondary outcome was TCVD excluding peripheral artery disease (PAD), defined as incident CAD, ICVD, and procedures only.

### 2.6 Machine learning techniques

Models were developed using four machine learning techniques—K-nearest neighbors (KNN), Logistic Regression, Random Forest, and Neural Networks—and were trained on two cohorts. One set of models was trained on a mixed cohort of patients with and without HIV, while the other set was trained exclusively on patients with HIV.

Categorical variables were encoded using one-hot encoding prior to model training. Continuous variables for the KNN model were normalized using Min-Max scaling to transform features to a range between 0 and 1, while Logistic Regression and Neural Network models used normalization based on the median and interquartile range to reduce sensitivity to outliers. Techniques to address class imbalance were not applied, as the outcome class frequency within the study population (∼20%) was considered sufficient for model training.

For the KNN model, which classifies a given input based on the proportion of outcome classes among its ‘k’ most similar neighbors, all normalized features contributed equally to distance calculations. The value of ‘k’ was optimized by testing increments of 10 between 40 and 500, selecting the value that yielded the highest area under the receiver operating characteristic curve (AUC). In the Logistic Regression model, input variables were passed through a sigmoid function to estimate the probability of ASCVD development. For the Random Forest model, we tuned the number of trees and the maximum tree depth by using randomized hyperparameter search and selecting the pair that produced the highest discrimination. This model also provided estimates of the relative importance of input features in predicting ASCVD.

The Neural Network models consisted of four layers and were trained up to 100 epochs with early stopping applied for a stable loss after 10 epochs. Models consisted of three fully connected hidden layers with 256, 128, and 64 units. L2 kernel regularization (with a coefficient of 1e-4) was applied to all hidden layers to help reduce overfitting by penalizing large weights. Batch normalization was applied after each hidden layer, and ReLU activation functions were applied after the first two hidden layers. The final output layer contained a sigmoid activation function, producing a probability between 0 and 1. Batch size was set to 32, and training was performed with a fixed learning rate without decay. The model was trained using the Adam optimizer with a learning rate of 0.001 and a binary cross-entropy loss function. Hyperparameters, including learning rate and L2 regularization coefficient, were tuned using a grid search method. Layer size and hyperparameters were tuned by evaluating alternative network widths and selecting the architecture that maximized discriminative performance on the validation set. Models were developed using Python 3.10.7, TensorFlow Version 2.20.0, and a random seed of 94.

### 2.7 Statistical analysis

Performance of the machine learning models was compared to the performance of the Framingham Risk Score (FRS) 10-year model [8] and the Pooled Cohort Equations (PCE) model [9]. Notably, the PCE model has narrower age eligibility criteria compared to FRS and machine learning models, as it was only validated for patients aged 40 to 79 years old. As a result, the comparator group for the PCE analysis was smaller.

To assess model performance, we evaluated both discrimination and calibration. Discrimination, the ability of a model to distinguish between individuals who did and did not develop ASCVD, was assessed using the area under the receiver operating characteristic curve (AUC), along with its 95% confidence interval, calculated using DeLong's method [10].

Calibration, the agreement between the predicted risk and the actual observed event rate, was assessed using three metrics: the Brier Score to measure overall error, the Hosmer-Lemeshow (H-L) test to evaluate goodness-of-fit, and a direct comparison between overall observed and predicted (expected) event rates. The Brier score is defined as the mean squared difference between the observed value of a binary outcome and its predicted probability. Brier scores closer to 0 indicate better calibration and scores closer to 1 indicate worse calibration. The H-L test assesses the goodness-of-fit of a model by comparing predicted probability of events with the observed event rate across deciles of risk groups, with a higher H-L score indicating greater departure. For the calculations in this study, a H-L test score of greater than 15.5 indicates a lack of good fit (i.e., significant difference between the observed and predicted values) at a statistical significance (alpha) level of 0.05. We calculated the ratio of observed (O) to expected (E) events (O:E ratio) as an additional measure of calibration, with a ratio close to 1 indicating good agreement.

We used 5-fold cross-validation to ensure model consistency. The training data was split randomly into five subsets, with each model trained on four folds—consisting of 80% of the dataset, which was then further partitioned into training (80%) and validation (20%) for the Neural Network and Logistic Regression models—and tested on the remaining one—consisting of 20% of the dataset—rotating through all folds to obtain average discrimination and calibration across the iterations.

### 2.8 Feature importance

For the Random Forest models, we utilized Gini impurity index to identify the most important features. In tree-based models, including Random Forests, the Gini impurity index is used to quantify feature importance based on the extent to which each feature decreases node impurity, which reflects how well a given decision in a decision tree partitions the data based on the outcome of interest. Additionally, permutation importance analyses were performed using 30 repeats to further evaluate feature importance robustness. Permutation importance quantifies the contribution of each feature by measuring the decrease in model performance after randomly shuffling the values of that feature. Accordingly, for each algorithm, we ordered the features by their importance for each of the five folds that we ran the Random Forest model. Finally, we summed their ranks across each fold and sorted them in ascending order based on their importance scores.

### 2.9 Handling of missing data

Missingness was assessed descriptively and was largely attributable to missing laboratory and biophysical data. The most common missing variables from the analysis were cholesterol measurements (33%), weight measurements (19%), and blood pressure measurements (17%). HIV patients were significantly less likely to have missing data compared to non-HIV patients, likely reflecting the closer and more frequent clinical follow-up that HIV patients experience compared those without HIV (Supplement Table 1). However, there was minimal correlation between missingness and measured variables (other than HIV status) suggesting that the unavailable data was missing completely at random or missing at random (Supplement Table 2). Although imputation strategies were considered, complete-case analysis was ultimately used, as the size of the remaining data afforded sufficient power for analysis, and because the primary aim was comparative model evaluation rather than development of a generalizable, deployable clinical prediction tool.

## 3 Results

### 3.1 Baseline demographics

Our original dataset included 274,004 Veterans both with and without HIV, of which 86,205 excluded due to pre-existing ASCVD or having age out of 20-79 years range. After excluding 64,495 patients with missing information,—123,304 Veterans were included in the study. Of these, 20,650 (17%) had HIV, and 102,654 (83%) did not have HIV. Individuals with HIV were, on average, slightly younger (51.7 years) than those without HIV (52.7 years).

The overall cohort was predominantly male (>95%). In terms of race, a smaller proportion of the HIV group was White (42.9%) compared to the non-HIV group (58.7%). Average HDL, total cholesterol, LDL, systolic blood pressure (SBP) levels, and smoking rates were comparable between the HIV and non-HIV groups. Rates of diabetes and hypertension were notably lower in the HIV group compared to the non-HIV group, with high standardized mean differences. Specifically, 15.7% of individuals with HIV had diabetes and 48.6% had hypertension, versus 30.6% and 67.3%, respectively, in the non-HIV group. Demographic data for the total cohort and the HIV and non-HIV subgroups are provided in Table 1.

**Table 1:** Baseline demographic and clinical characteristics of the study population by HIV status.

Variable	All Mean (SD) or %	HIV Mean (SD) or %	Non-HIV Mean (SD) or %	Standardized Mean Difference	HIV Only Model Mean (SD)∗ or %
N Available	123304	20650	102654	-	16717
Age	52.6 (10.8)	51.7 (10.9)	52.7	0.14	51.5
Male	95.9%	96.5%	95.8%	0.03	96.7%
White	56.1%	42.9%	58.7%	0.32	42.6%
Black	35.7%	50.7%	46.9%	0.37	50.0%
Average HDL-c	45.2 (15.0)	44.5 (16.1)	45.3 (14.7)	0.07	44.2 (16.0)
Total Cholesterol (mg/dl)	181.5 (41.9)	177.1 (41.7)	182.4 (41.9)	0.17	176.9 (42.0)
Average LDL-c (mg/dl)	107.2 (35.2)	102.2 (34.4)	108.2 (35.2)	0.24	101.9 (34.7)
Average SBP mmHg	130.4 (13.9)	128.1 (14.0)	130.8 (13.9)	0.27	128.0 (13.7)
Diabetes	28.1%	15.7%	30.6%	0.35	15.4%
Smoking	44.6%	44.9%	44.6%	0.00	45.0%
Hypertension	64.1%	48.6%	67.3%	0.37	48.3%
Hypertension Medication	53.7%	39.5%	56.5%	0.34	39.8%
Protease Inhibitor	-	-	-	-	40.4%
Percent on ART Therapy	-	-	-	-	87.1%
Average CD4	-	-	-	-	516.1 (310.0)
Viral load (median, IQR)	-	-	-	-	48.0 (20.0, 1035.0)

ART: antiretroviral therapy; HDL-c: high-density lipoprotein cholesterol; IQR: inter-quartile range; LDL-c: low-density lipoprotein cholesterol; SBP: systolic blood pressure; SD: standard deviation.

Over a mean follow-up time of 8.5 years (SD = 2.3 years), ASCVD events occurred among 4224 HIV patients, including 2618 CAD diagnoses, 1395 ICVD diagnoses, 1166 PAD diagnoses, and 627 procedural interventions. Among non-HIV patients, 23,213 individuals experienced an ASCVD event, including 14,817 CAD diagnoses, 7115 ICVD diagnoses, 6753 PAD diagnoses, and 2919 procedural interventions. The incidence of the primary ASCVD outcome was 20.4% among Veterans with HIV and 22.3% among those without HIV, despite the known association between HIV and elevated cardiovascular risk. The corresponding incidence for the secondary outcome (excluding PAD) was 17.5% among Veterans with HIV and 18.9% in the overall population.

### 3.2 Model discrimination

Model discrimination and 95% confidence intervals (CI) are given for all models in Table 2. Overall, the models showed fair to good discrimination. Machine learning models trained on patients with and without HIV had an equivalent or better performance compared to the models of the same type that were trained just on patients with HIV.

**Table 2:** Comparison of discrimination ability of machine learning models with traditional models in predicting the primary^a^ cardiovascular disease outcome.

	Discrimination: Area Under ROC Curve (95% Confidence Interval)		
	Existing Models	Machine Learning Models	
		Trained on All Data	Trained on HIV-only Data
Framingham Risk Score Model	0.661(0.642–0.680)	-	-
Pooled Cohort Equations Model	0.652(0.630–0.673)	-	-
K-Nearest Neighbors Model	-	0.640(0.620–0.660)	0.571 (0.547–0.595)
Random Forest Model	-	0.707 (0.688–0.726)	0.707 (0.686–0.728)
Logistic Regression Model	-	0.701 (0.683 –0.720)	0.643 (0.618–0.667)
Neural Network Model	-	0.707 (0.688–0.726)	0.663 (0.640–0.685)

a The primary cardiovascular disease outcome is coronary artery disease, ischemic cerebrovascular disease, and peripheral artery disease.

**Fig. 1:**
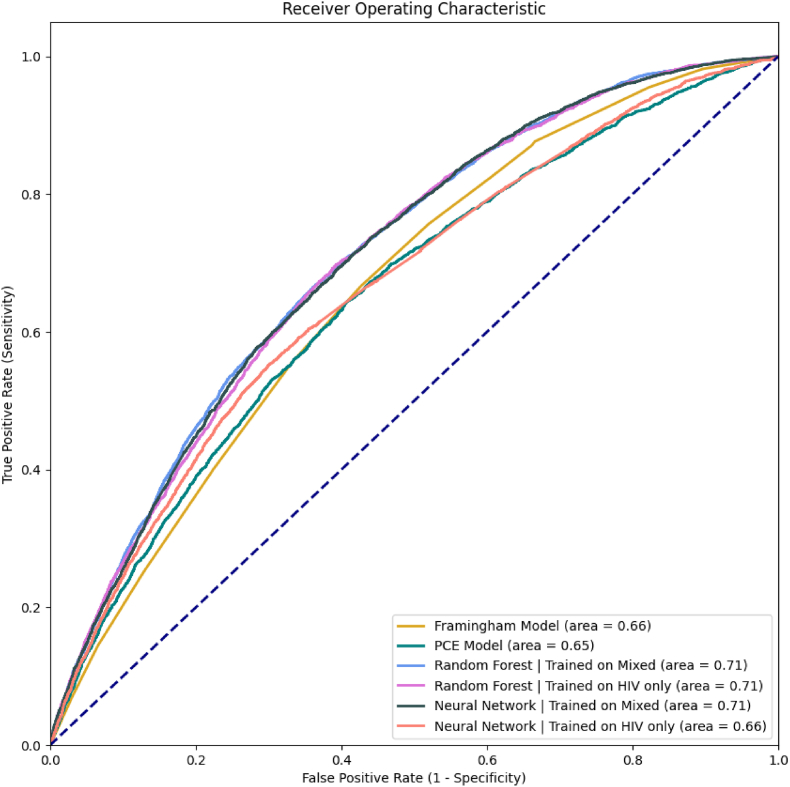
Area under receiver operator curve in predicting the primary∗ cardiovascular disease outcome comparing traditional and two machine learning models. ∗The primary cardiovascular disease outcome is coronary artery disease, ischemic cerebrovascular disease, and peripheral artery disease. PCE – pooled cohort equation.

For the existing risk models predicting the primary outcome, the AUC was 0.66 for the FRS model and 0.65 for the PCE model. When comparing machine learning approaches to established models, the Random Forest, Logistic Regression, and Neural Networks demonstrated higher discrimination. The Random Forest (0.707 [0.688–0.726]), Neural Network (0.707 [0.688–0.726]), and Logistic Regression (0.701 [0.683–0.720]) models trained on all data achieved statistically significant improvements compared to both the PCE model and FRS models. The Random Forest model trained on HIV-only data (0.707 [0.686–0.728]) similarly showed incremental improvement relative to both existing models. The remaining models trained on HIV-only data did not show any significant improvement. Additionally, the K-Nearest Neighbors model had lower discrimination overall (0.64) and performed especially poorly when trained only on individuals with HIV (0.57). Comparisons of AUC curves between extant models, Neural Network models, and Random Forest models are shown in Fig. 1.

For the secondary outcome, the AUC was 0.66 for the FRS model and 0.64 for the PCE model. The Random Forest (0.695 [0.675–0.715]) and Neural Network (0.696 [0.676–0.716]) models trained on all data similarly showed statistically significant improvement compared to both models. The Random Forest model trained on HIV-specific data (0.691 [0.669–0.713]) and the Logistic Regression (0.692 [0.672–0.712]) model trained on all data showed moderate improvements compared to the PCE model only. On the otherhand, KNN, the Neural Network and Logistic Regression models trained on HIV-specific data did not show improvement over the traditional models (Supplement Table 3 and **Supplement Figure**).

### 3.3 Model calibration

Calibration metrics for traditional and ML models are summarized in Table 3. For all models, the Brier score ranged between 0.149 and 0.183. Both Random Forest models and the Neural Network model trained on mixed data achieved the lowest Brier scores (0.149 and 0.149 respectively), suggesting the most accurate and well-calibrated predictions. In contrast, traditional models like the FRS and PCE had slightly higher Brier scores at 0.167 and 0.178. Notably, Logistic Regression models trained only on HIV-positive individuals had the highest Brier scores (up to 0.183). Brier scores of models trained on mixed HIV and non-HIV populations were lower than those of models trained exclusively on HIV.

**Table 3:** Comparison model calibration between machine learning and traditional models in predicting the primary^a^ cardiovascular disease outcome.

	Brier Score	Hosmer-Lemeshow (p-value)	Expected Event Rate	Observed Event Rate	O:E Ratio
Framingham Risk Score Model	0.167	1032.2 (<0.01)	1999.1	4224	2.113
Pooled Cohort Equations Model	0.178	494.2 (<0.01)	2356.8	4097	1.738
K-Nearest Neighbors Model - trained on all data	0.158	16.3 (0.06)	4361.8	4224	0.968
K-Nearest Neighbors Model - trained on HIV only	0.162	13.5 (0.14)	3373.4	3431	1.017
Random Forest Model - trained on all data	0.149	18.1 (0.03)	4122.7	4224	1.025
Random Forest Model - trained on HIV only	0.149	15.5 (0.08)	3459.8	3431	0.992
Logistic Regression Model - trained on all data	0.153	106.0 (<0.01)	3809.9	4224	1.109
Logistic Regression Model - trained on HIV only	0.183	Undefined	2573.6	3431	1.333
Neural Network Model - trained on all data	0.149	27.3 (<0.01)	4169.8	4224	1.013
Neural Network Model - trained on HIV only	0.155	48.0 (<0.01)	3315.4	3431	1.035

a The primary cardiovascular disease outcome is coronary artery disease, ischemic cerebrovascular disease, and peripheral artery disease.

The FRS and PCE models had poor calibration, with high H-L statistic values (1032.2 for FRS and 494.2 for PCE). In contrast, machine learning models—particularly non-layered models of K-Nearest Neighbors and Random Forest—showed much lower H-L values, with some H-L statistics falling below the 15.5 threshold for statistical significance. The Logistic Regression models trained only on HIV-positive patients yielded undefined H-L values, likely due to extreme predicted probabilities within some deciles.

**Fig. 2:**
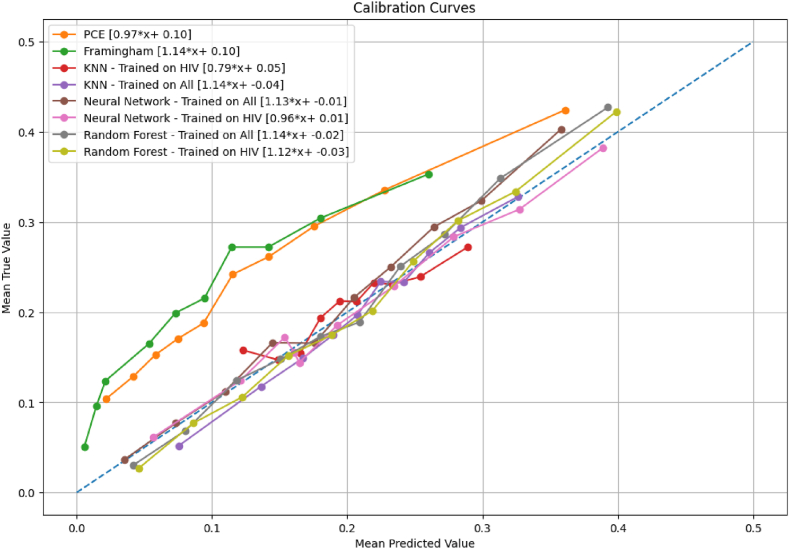
Calibration plot comparing predicted versus observed risk for the primary∗ cardiovascular disease outcome across traditional risk models and machine learning models ∗The primary cardiovascular disease outcome is coronary artery disease, ischemic cerebrovascular disease, and peripheral artery disease. PCE – pooled cohort equation. KNN – K-nearest neighbors.

Additionally, the FRS and PCE models showed substantial underprediction for the primary ASCVD outcome, with O:E ratios around 2.1 and 1.7, respectively. In contrast, the O:E ratios were very close to 1.0 for the ML models, especially for the Random Forest, Neural Networks, and KNN models (Table 3). Calibration plots with slope and intercept for the aforementioned models are shown in Fig. 2. The findings were comparable for the secondary outcome (Supplement Table 4).

### 3.4 Feature importance

We utilized Gini impurity and permutation importance to identify the most important features in predicting the primary outcome when training and testing the Random Forest models (Table 4). According to the Gini impurity feature importance analysis, age, systolic blood pressure, weight, triglycerides, LDL, HDL, total cholesterol, diastolic blood pressure, height, and hypertension ranked in the top 10 important features. Prescribed hypertension medications, diabetes mellitus, and BMI also ranked higher in feature importance. Models derived using HIV-specific data placed a greater importance on CD4 count and HIV viral load. Findings for the Gini impurity were comparable for the secondary outcome (Supplement Table 5).

**Table 4:** Rank list of variable feature importance in predicting the primary^a^ cardiovascular disease outcome in the Random Forest machine learning models.

Rank	Trained on All Data		Trained on HIV-only Data	
	Gini Impurity	Permutation Importance	Gini Impurity	Permutation Importance
1	Age	Age	Age	Age
2	Systolic Blood Pressure	Diabetes Mellitus	Systolic Blood Pressure	Diabetes Mellitus
3	Weight	Diabetes Mellitus Complications	Triglycerides	Hypertension Medications
4	Triglycerides	Systolic Blood Pressure	CD4 Count	Hypertension
5	LDL-c	Smoking	Weight	Systolic Blood Pressure
6	HDL-c	Pulmonary Disease	LDL-c	Viral Load
7	Total Cholesterol	Hypertension Medications	Total Cholesterol	Pulmonary Disease
8	Diastolic Blood Pressure	Hypertension	HDL-c	Diastolic Blood Pressure
9	Height	HDL-c	Diastolic Blood Pressure	Diabetes Mellitus Complications
10	Hypertension	Valvular Disease	Height	Smoking
11	Diabetes Mellitus	Statin Medication	Viral Load	Valvular Disease
12	Hypertension Medications	Triglycerides	Hypertension	Statin Medication
13	Diabetes Mellitus Complications	Diastolic Blood Pressure	Hypertension Medications	Protease Inhibitor
14	BMI	Hypertension Complications	BMI	Triglycerides
15	Pulmonary Disease	Needs Fluids/Electrolyte Abnormalities	Diabetes Mellitus	HDL-c

BMI: Body mass index; HDL-c: high-density lipoprotein cholesterol; LDL-c: low-density lipoprotein cholesterol.

a The primary cardiovascular disease outcome is coronary artery disease, ischemic cerebrovascular disease, and peripheral artery disease.

Permutation importance analysis similarly identified age, blood pressure and hypertension-related variables, and diabetes mellitus as influential predictors across models, with a greater importance on smoking and a lesser importance on lipid-related factors. In the HIV-specific models, HIV viral load also demonstrated increased importance, alongside protease inhibitor use.

## 4 Discussion

To the best of our knowledge, this is the first study to create and compare machine learning models for cardiovascular risk prediction specifically in people living with HIV (PLHIV). Our findings demonstrate that machine learning approaches, particularly Random Forest, Neural Networks, and Logistic Regression, modestly outperformed traditional risk models such as the FRS and PCE, especially when trained on the full dataset of people with and without HIV. In the era of widespread antiretroviral therapy (ART) and increased longevity among PLHIV, our results suggest potential for machine learning to support more personalized and effective cardiovascular care.

Traditional cardiovascular risk models such as the FRS and PCE were developed in general population cohorts that may be limited in reflecting the elevated baseline risk in PLHIV [4]. These models apply algorithms with capped contributions from traditional risk factors, which can underestimate true event probability in high-risk subgroups. Additionally, built-in risk ceilings for these models limit their predictive power for populations with higher rates of ASCVD events, as in this study. As a result, individuals from populations with non-traditional risk factors, such as inflammation or adverse social determinants of health, may be categorized as low or intermediate risk, despite a significantly higher observed incidence of ASCVD events. A recent study using the Research Network of Integrated Clinical Systems (CNICS) data reported even more significant ASCVD risk underestimation in PLHIV when using the American Heart Association's newest risk calculator: Predicting Risk of Cardiovascular Disease Events (PREVENT), with an O:E ratio of 2.69 [11]. Another prospective cohort study leveraging data from the REPRIEVE trial found variable calibration in different subgroups of PLHIV, highlighting the need for further research to develop risk scores accurate in predicting ASCVD among PLHIV in multiple risk strata [12]. Machine learning algorithms are uniquely suited to capturing nonlinear and high-order interactions between variables. In the context of HIV-associated ASCVD, where traditional risk factors converge with immunologic, virologic, and treatment-related drivers, machine learning enables the detection of nuanced patterns and synergistic effects that conventional models overlook. This analytic flexibility facilitates a more biologically grounded and individualized estimation of cardiovascular risk, particularly in patients with heterogeneous clinical risk profiles.

In this study, models trained exclusively on HIV-positive populations underperformed relative to mixed-population models, despite incorporating HIV-specific variables. This likely reflects reduced sample size and loss of cross-population structure and loss of between-group variation in risk profiles present in the full cohort. However, at the same time, feature importance analyses from the Random Forest models identified traditional cardiovascular risk factors, such as age, blood pressure, diabetes mellitus, and lipid measures as the strongest predictors of ASCVD risk, with the different feature-ranking algorithms showing variations in the relative ranking of specific predictors but consistently emphasizing the central role of established cardiometabolic risk factors across approaches. This suggests that well-established cardiovascular risk factors in the general population continue to play a dominant role in ASCVD risk among PLHIV, aligning with findings from Rosenson et al. that clinical risk scores developed for the general population remain predictive in this population [13]. Differences between Gini-based and permutation importance likely reflect their underlying methods, with Gini importance favoring variables with higher variability, while permutation importance may underestimate the contribution of variables that are highly collinear with others, such as LDL cholesterol and total cholesterol.

In addition to traditional risk factors, feature importance analysis from the Random Forest models highlighted HIV-specific variables, such as CD4 count, viral load, and protease inhibitor use, as prominent predictors in models trained exclusively on HIV-positive patients. As viral load and CD4 count often correlate with immune modulation and HIV control, our findings potentially reflect the contribution of chronic immune activation and systemic inflammation to ASCVD pathogenesis in PLHIV, which may not be fully captured by traditional metabolic risk factors [14]. Protease inhibitor exposure may additionally reflect treatment–related metabolic effects known to influence cardiometabolic risk. This result also highlights that the level of HIV control was a key contributor to cardiovascular risk prediction in this population.

Additionally, most machine learning models exhibited superior calibration compared to FRS and PCE, with lower H-L scores and more favorable O:E event ratios, indicating more accurate population-level risk estimation. Particularly, Random Forest and Neural Network models trained on the full cohort achieved the best discrimination along with improved calibration, while FRS and PCE consistently underpredicted ASCVD risk in the HIV subgroup. This miscalibration of the traditional models was further demonstrated in the calibration curve analysis, as FRS and PCE showed markedly elevated calibration intercepts with systematic underestimation of observed ASCVD risk across all predicted risk ranges. Consistent with prior studies, this study highlights the critical gap in current clinical practice that could be addressed by using machine learning models, where traditional models may not identify some proportion of PLHIV who would benefit from earlier initiation of primary CVD prevention measures [4].

Interestingly, despite the established association between HIV and elevated cardiovascular risk, the crude incidence of ASCVD events was slightly lower in the HIV cohort than in the non-HIV cohort. This likely reflects differences in baseline clinical characteristics, competing mortalities, and residual confounding, underscoring that unadjusted event rates may not fully capture the relative cardiovascular risk attributable to HIV status.

In recent years, growing research has been directed toward identifying and mitigating cardiovascular risk in high-risk subpopulations of PLHIV. The results of the REPRIEVE trial showed that pitavastatin therapy for PLHIV with low to moderate risk of ASCVD significantly lowered risk of major adverse cardiovascular events [15]. Similarly, the SMART study showed that interruption of ART in PLHIV increased risk of adverse cardiovascular events [16]. Effective cardiovascular prevention in PLHIV requires accurate risk stratification to ensure that individuals who may benefit from initiation of statin therapy and intensification of risk factor modification are managed appropriately. The superior predictive performance of the machine learning models developed in this study suggests a meaningful advance over conventional tools. These models provide a promising pathway to improve risk stratification, thereby enabling proactive implementation of preventive strategies, including lipid-lowering therapy and intensive risk factor modification.

However, while machine learning models such as Random Forests and Neural Networks demonstrated superior predictive accuracy, their relative complexity presents challenges for clinical adoption. These models often function as "black boxes," providing limited interpretability regarding how predictions are derived, an important consideration for clinicians making risk-based treatment decisions. In contrast, more interpretable models like Logistic Regression offer greater transparency and are more readily integrated into clinical workflows, albeit at the expense of some predictive power. Balancing these trade-offs requires careful consideration of the intended clinical use. The development of hybrid approaches, such as simplified risk scores derived from high-performing machine learning models or the use of ensemble methods, may offer a translatable and pragmatic path forward, combining predictive strength with operational feasibility.

While this study offers many important insights, it also has several limitations that highlight areas for further investigation. Because the dataset is derived from the Veterans Health Administration, which predominantly serves a male population, the findings may not be generalizable to women or non-Veteran populations. The overwhelming male predominance in the cohort may introduce gender-based bias in model development and performance, limiting the ability of the models to accurately capture cardiovascular risk patterns that differ by sex. Approximately 95% of the study cohort consisted of male Veterans, representing a major limitation that restricts the broader generalizability of these findings to more diverse patient populations. Future work in more heterogeneous cohorts will be essential to determine the external validity and equity of these models across gender-balanced and non-Veteran populations.

Furthermore, excluding patients with missing variables may have introduced selection bias and further limited generalizability. While missing data was excluded because the study aimed to test how the machine learning techniques compared to the existing risk models, this approach may have introduced selection bias by preferentially excluding patients with incomplete data who may differ systematically in clinical characteristics and risk profiles from those with complete records, such as by having more frequent healthcare follow-up.

Finally, an important limitation from a translational perspective is the absence of external validation, as all model performance results were derived from internal validation alone. Without validation in independent external cohorts, the clinical applicability and generalizability of these models remain uncertain. Additional research is therefore needed to externally validate cardiovascular risk prediction models across broader and more diverse clinical settings for PLHIV. Future directions should focus on validating these programmatic approaches in external datasets that include different patient populations, particularly women and non-Veterans, to assess model robustness. Further subgroup analyses—such as stratifying by age or presupposed risk based on extant models—could potentially improve model performance within specific populations. These are of particular interest given the key demographic differences in risk factors, notably diabetes and hypertension, observed between the HIV and non-HIV groups in this study. Future developers may also seek to incorporate more advanced data metrics, like more specific ART medications and classes with time-updated or cumulative exposure measures, treatment adherence, polygenic risk scores, or other comorbid HIV-related infections, into their models to determine whether they further enhance model performance beyond what was demonstrated in this study [17].

## 5 Conclusion

Machine learning approaches, particularly Random Forest, Neural Networks, and Logistic Regression, offer a modest but meaningful improvement over traditional risk models in terms of discrimination and calibration, especially when trained on combined datasets that include both HIV and non-HIV populations. Well-established cardiovascular risk factors continue to play a dominant role in ASCVD risk among PLHIV, while HIV-specific variables help to enhance model performance. Future studies in more diverse populations should validate and refine these models for ASCVD prevention in PLHIV, paving the way for pragmatic implementation and eventual clinical use.

## Access to data and data analysis

Hari Dandapani and Sebhat Erqou had full access to all the data in the study and took responsibility for the integrity of the data and the accuracy of the data analysis. Hari Dandapani, Vrishali Lopes, Christopher Halladay, Sebhat Erqou, Wen-Chih Wu, and James Rudolph (Providence VA and Brown U) were involved in the data acquisition and analysis for this manuscript.

## Data availability statement

The data used in this study were obtained from the United States Department of Veterans Affairs (VA) Veterans Health Administration (VHA) electronic health records. These data contain protected health information and are not publicly available due to federal privacy regulations and VA data use policies. Access to VA data requires an approved research protocol, Institutional Review Board (IRB) approval, and authorization through the VA Informatics and Computing Infrastructure (VINCI). Investigators who meet these criteria may request access through the VA research data access process. Analytical code supporting the findings of this study is available from the corresponding author upon reasonable request, subject to VA data security requirements.

## Funding sources

The research reported/outlined here was supported by the 10.13039/100000738Department of Veterans Affairs, Veterans 10.13039/100018696Health Administration, VISN 1 Career Development Award to SE. SE is also funded by Center for AIDS Research, The 10.13039/100014082Rhode Island Foundation, Lifespan Cardiovascular Institute, and Mary Washington Hospital. 10.13039/100016335JLR, 10.13039/100015519WW, SE, and 10.13039/100028447JS are funded by the 10.13039/100000738VA 10.13039/100007217Health Services Research and Development Center of Innovation in Long Term Services and Supports (CIN 13-419; C19-20-213). 10.13039/100004792HD was supported in part by NIH/10.13039/100000060NIAID under R25AI140490.

The views expressed in this article are those of the authors and do not necessarily reflect the position or policy of the Department of Veterans Affairs or the United States government.

## CRediT authorship contribution statement

**Hari Dandapani:** Formal analysis, Methodology, Writing – original draft. **Yi-Yun Chen:** Conceptualization, Writing – review & editing. **Michael Kwok:** Conceptualization, Writing – review & editing. **Vrishali Lopes:** Data curation, Methodology. **Christopher Halladay:** Data curation, Methodology. **Gerald S. Bloomfield:** Conceptualization, Writing – review & editing. **Christoper T. Longenecker:** Conceptualization, Writing – review & editing. **Jennifer L. Sullivan:** Conceptualization, Writing – review & editing. **Gaurav Choudhary:** Conceptualization, Writing – review & editing. **James L. Rudolph:** Conceptualization, Funding acquisition, Supervision, Writing – review & editing. **Wen-Chih Wu:** Conceptualization, Funding acquisition, Supervision, Writing – review & editing. **Sebhat Erqou:** Conceptualization, Methodology, Supervision, Visualization, Writing – review & editing.
