# Supplementary material for: Developing machine learning models to improve cardiovascular risk prediction for people living with HIV
Source: Int J Cardiol Cardiovasc Risk Prev. 2026 Jul 18;30:200683. doi: 10.1016/j.ijcrp.2026.200683 (PMC13396610; doi:10.1016/j.ijcrp.2026.200683)
Supplement: Multimedia component 1 [file mmc1.docx]

**Supplement Material**

**Developing Machine Learning Models to Improve Cardiovascular Risk Prediction for People Living With HIV**

**Page 2.** **Supplement Figure.** Area under receiver operator curve in predicting the secondary cardiovascular disease outcome comparing traditional and two machine learning models.

**Page 3.** **Supplement Table 1:** Missingness of Clinical and Laboratory Variables by HIV Status.

**Page 4. Supplement Table 2:** Pairwise Correlation Analysis of Missingness Indicators Across Clinical Variables.

**Page 5. Supplement Table 3:** Comparison of discrimination ability of machine learning models with traditional models in predicting the secondary cardiovascular disease outcome.

**Page 6. Supplement Table 4:** Comparison model calibration between machine learning and traditional models in predicting the secondary cardiovascular disease outcome.

**Page 7.** **Supplement Table 5:** Rank list of variable feature importance in predicting the secondary cardiovascular disease outcome in the random forest machine learning model.

**Supplement Figure.** Area under receiver operator curve in predicting the secondary* cardiovascular disease outcome comparing traditional and two machine learning models.


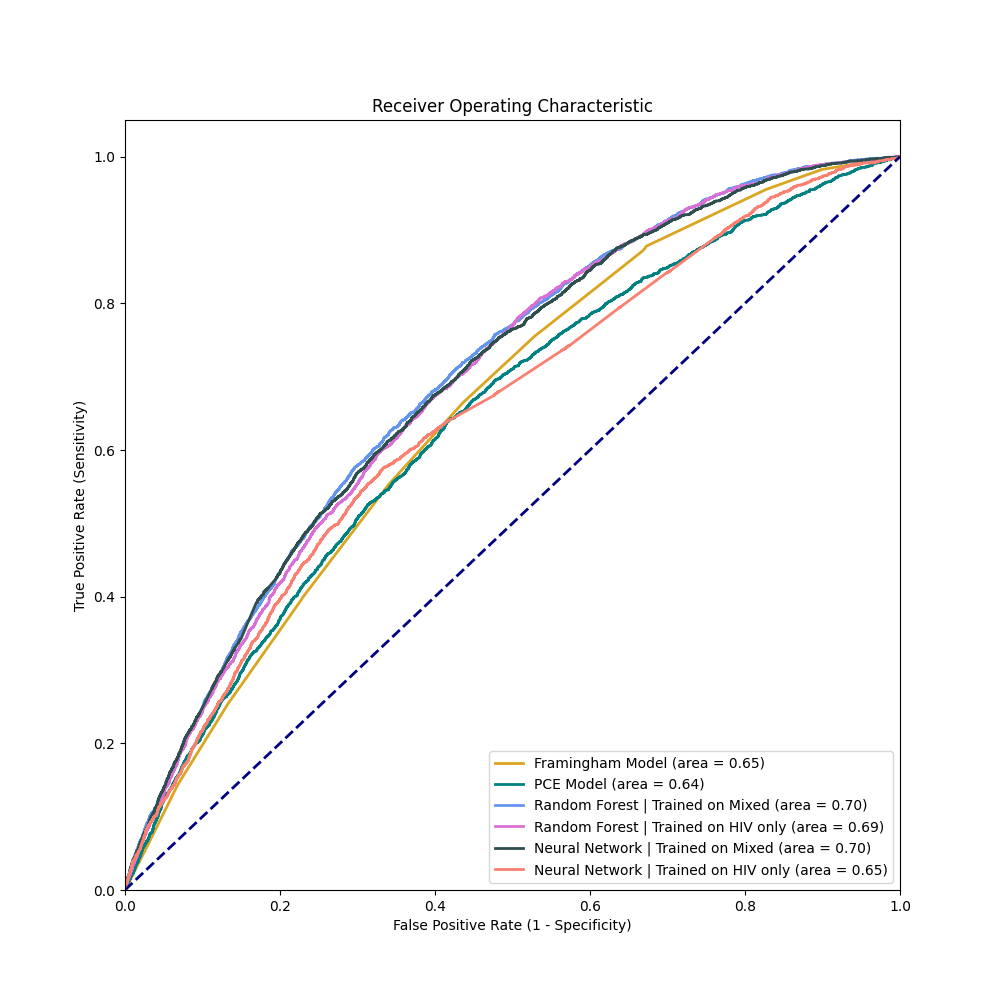


*The secondary cardiovascular disease outcome is coronary artery disease and ischemic cerebrovascular disease.

**Supplement Table 1:** Missingness of Clinical and Laboratory Variables by HIV Status.

| **Variable** | **All Participants** | | **Non-HIV Participants** | | **Participants with HIV** | | **P-value** |
| --- | --- | --- | --- | --- | --- | --- | --- |
|  | **N Available** | **% Missing** | **N Available** | **% Missing** | **N Available** | **% Missing** |  |
| **Height** | **186412** | **0.7%** | **159646** | **0.8%** | **26766** | **0.3%** | **<0.001** |
| **Weight** | **157316** | **16.2%** | **131006** | **18.6%** | **26310** | **2.0%** | **<0.001** |
| **BMI** | **157220** | **16.3%** | **130960** | **18.6%** | **26260** | **2.2%** | **<0.001** |
| **SBP** | **160112** | **14.7%** | **133626** | **17.0%** | **26486** | **1.3%** | **<0.001** |
| **DBP** | **160105** | **14.7%** | **133619** | **17.0%** | **26486** | **1.3%** | **<0.001** |
| **LDL** | **130242** | **30.6%** | **108670** | **32.5%** | **21572** | **19.6%** | **<0.001** |
| **HDL** | **130263** | **30.6%** | **108641** | **32.5%** | **21622** | **19.4%** | **<0.001** |
| **Triglycerides** | **129917** | **30.8%** | **108260** | **32.7%** | **21657** | **19.3%** | **<0.001** |
| **Total Cholesterol** | **129623** | **31.0%** | **107895** | **33.0%** | **21728** | **19.0%** | **<0.001** |
| **Ethnicity** | **179328** | **4.5%** | **153013** | **4.9%** | **26315** | **2.0%** | **<0.001** |

**Supplement Table 2.** Correlation Between Missingness Indicators and Clinical Variables.

|  | Height | Weight | BMI | SBP | DBP | LDL | HDL | TG | TC |
| --- | --- | --- | --- | --- | --- | --- | --- | --- | --- |
| Height_m | NaN | -0.006 | NaN | -0.004 | -0.006 | -0.001 | 0.001 | -0.001 | 0.001 |
| Weight_m | 0.004 | NaN | NaN | 0.036 | 0.04 | 0.014 | 0 | -0.001 | 0.011 |
| BMI_m | 0.004 | -0.006 | NaN | 0.035 | 0.038 | 0.013 | 0 | -0.001 | 0.01 |
| SBP_m | 0.004 | 0.003 | 0.003 | NaN | NaN | 0.014 | 0 | 0.003 | 0.013 |
| DBP_m | 0.004 | 0.003 | 0.003 | 0.002 | NaN | 0.014 | 0 | 0.003 | 0.013 |
| LDL_m | 0.007 | -0.065 | -0.073 | -0.014 | 0.012 | NaN | -0.03 | 0.142 | 0.014 |
| HDL_m | 0.007 | -0.066 | -0.073 | -0.014 | 0.01 | 0.001 | NaN | 0.037 | -0.003 |
| TG_m | 0.007 | -0.065 | -0.072 | -0.014 | 0.011 | 0.001 | -0.006 | NaN | -0.003 |
| TC_m | 0.007 | -0.063 | -0.069 | -0.013 | 0.007 | 0.006 | -0.007 | -0.007 | NaN |

Heatmaps display Pearson correlation coefficients between binary missingness indicators (1 = missing, 0 = observed) for each clinical variable and the corresponding set of observed patient characteristics.

BMI – body mass index; SBP – systolic blood pressure; DBP – diastolic blood pressure; LDL – low density lipoprotein; HDL – high density lipoprotein; TG – triglycerides; TC – total cholesterol.

“_m” is a missingness indicator

NaN indicates that there was no calculable variance in the clinical variable between missing and non-missing cohorts.

**Supplement Table 3:** Comparison of discrimination ability of machine learning models with traditional models in predicting the secondary* cardiovascular disease outcome.

| Model | Discrimination: Area Under ROC Curve (95% Confidence Interval) | | |
| --- | --- | --- | --- |
|  | Existing Models | Machine Learning Models | |
|  |  | Trained on All Data | Trained on HIV-only Data |
| Framingham Risk Score Model | 0.655 (0.634–0.675) | - | - |
| Pooled Cohort Equations Model | 0.642(0.620–0.665) | - | - |
| K-Nearest Neighbors Model | - | 0.633 (0.612–0.654) | 0.566 ( 0.541–0.591) |
| Random Forest Model | - | 0.695 (0.675–0.715) | 0.691 (0.669–0.713) |
| Logistic Regression Model | - | 0.692 (0.672–0.712) | 0.634 (0.609–0.659) |
| Neural Network Model | - | 0.696 (0.676–0.716) | 0.647 (0.623–0.671) |

*The secondary cardiovascular disease outcome is coronary artery disease and ischemic cerebrovascular disease.

**Supplement Table 4:** Comparison model calibration between machine learning and traditional models in predicting the secondary* cardiovascular disease outcome.

|  | Brier Score | Hosmer-Lemeshow  (p-value) | Expected Event Rate | Observed Event Rate | O:E Ratio |
| --- | --- | --- | --- | --- | --- |
| Framingham Risk Score Model | 0.145 | 644.8 (<0.01) | 1999.1 | 3615 | 1.808 |
| Pooled Cohort Equations Model | 0.157 | 287.5 (<0.01) | 2356.8 | 3509 | 1.489 |
|  |  |  |  |  |  |
| K-Nearest Neighbors Model - trained on all data | 0.140 | 12.5 (0.19) | 3693.3 | 3615 | 0.979 |
| K-Nearest Neighbors Model - trained on HIV only | 0.144 | 14.7 (0.10) | 2900.8 | 2938 | 1.013 |
|  |  |  |  |  |  |
| Random Forest Model - trained on all data | 0.135 | 14.0 (0.12) | 3517.8 | 3615 | 1.028 |
| Random Forest Model - trained on HIV only | 0.135 | 9.3 (0.41) | 2973.9 | 2938 | 0.988 |
|  |  |  |  |  |  |
| Logistic Regression Model - trained on all data | 0.137 | 73.8 (<0.01) | 3969.6 | 3615 | 0.911 |
| Logistic Regression Model - trained on HIV only | 0.180 | Undefined | 2901.4 | 2938 | 1.013 |
|  |  |  |  |  |  |
| Neural Network Model - trained on all data | 0.135 | 17.2 (0.05) | 3662.4 | 3615 | 0.987 |
| Neural Network Model - trained on HIV only | 0.141 | 30.2 (<0.01) | 2732.0 | 2938 | 1.075 |

*The secondary cardiovascular disease outcome is coronary artery disease and ischemic cerebrovascular disease.

**Supplement Table 5:** Rank list of variable feature importance in predicting the secondary* cardiovascular disease outcome in the Random Forest machine learning models.

| Trained on All Data | | | | Trained on HIV-only Data | | | |
| --- | --- | --- | --- | --- | --- | --- | --- |
| Rank | Variable | Rank | Variable | Rank | Variable | Rank | Variable |
| 1 | Age | 11 | Hypertension Medications | 1 | Age | 11 | Height |
| 2 | Systolic Blood Pressure | 12 | Diabetes Mellitus | 2 | Triglycerides | 12 | Hypertension |
| 3 | Triglycerides | 13 | BMI | 3 | Systolic Blood Pressure | 13 | Hypertension Medications |
| 4 | Weight | 14 | Diabetes Mellitus Complications | 4 | Weight | 14 | BMI |
| 5 | LDL-c | 15 | Pulmonary Disease | 5 | CD4 Coutn | 15 | Diabetes Mellitus |
| 6 | Total Cholesterol | 16 | Statin Medication | 6 | LDL-c | 16 | Pulmonary Disease |
| 7 | HDL-c | 17 | Smoking | 7 | Total Cholesterol | 17 | Protease Inhibitor |
| 8 | Diastolic Blood Pressure | 18 | Needs Fluids / Electrolyte Abnormalities | 8 | HDL-c | 18 | Smoking |
| 9 | Height | 19 | Anemia | 9 | Diastolic Blood Pressure | 19 | Statin Medication |
| 10 | Hypertension | 20 | Obesity | 10 | Viral Load | 20 | Anemia |

*The secondary cardiovascular disease outcome is coronary artery disease and ischemic cerebrovascular disease.

BMI: Body mass index; HDL-c: high-density lipoprotein cholesterol; IQR: inter-quartile range; LDL-c: low-density lipoprotein cholesterol
